# A Retrospective Study of Superficial Type Atypical Lipomatous Tumor

**DOI:** 10.3389/fmed.2020.609515

**Published:** 2020-12-17

**Authors:** Emi Mashima, Yu Sawada, Natsuko Saito-Sasaki, Kayo Yamamoto, Shun Ohmori, Daisuke Omoto, Haruna Yoshioka, Manabu Yoshioka, Etsuko Okada, Takatoshi Aoki, Masanori Hisaoka, Motonobu Nakamura

**Affiliations:** ^1^Department of Dermatology, University of Occupational and Environmental Health, Kitakyushu, Japan; ^2^Department of Radiology, University of Occupational and Environmental Health, Kitakyushu, Japan; ^3^Department of Pathology and Oncology, University of Occupational and Environmental Health, Kitakyushu, Japan

**Keywords:** atypical lipomatous tumor (ALT), magnetic resonance imaging (MRI), clinical characteristics, physical examination, tumor size

## Abstract

Atypical lipomatous tumor (ALT) has been defined as a well-differentiated liposarcoma exhibiting a higher frequency of a local recurrence after surgical resection. ALT is mainly classified into deep type and superficial type. Compared with deep type ALT, superficial type ALT is rarely observed. One of the most important issues is that little has been known about superficial type ALT and it is not easy to predict the presence of superficial type ALT before surgical resection. To clarify the clinical manifestations of superficial type ALT, we examined 15 cases with superficial type ALT and 118 cases with benign lipoma, and analyzed their differences in clinical characteristics and the findings of MRI test. In clinical characteristics, the tumor size of superficial type ALT was significantly greater than that of benign lipoma, and superficial type ALT showed a significantly higher frequency of the tumor size of more than 4 cm. Superficial type ALT exhibited poor tumor mobility and hardness with elastic soft. In addition, a significantly higher frequency of tumor location of superficial type ALT was observed in extremities. Among tumor sites at the trunk, buttocks, and shoulder were high frequent location in superficial type ALT. In an MRI examination, superficial type ALT exhibited a significantly higher frequency of the septal structures compared with benign lipoma. The combinations of clinical characteristics, including physical examinations, MRI, and histological examinations, are helpful for the diagnosis of superficial type ALT.

## Introduction

Adipose tissue is one of the sources of stem cells, which are derived from the embryonic mesenchyme. Because of its characteristics, adipose tissue sometimes develops into adipocytic tumors, such as lipoma, which are commonly seen in dermatological clinics. Among various adipocyte-derived tumors, Evans and colleagues reported a well-differentiated liposarcoma occurring in a subcutaneous location or within a muscle layer in 1979 (1). Furthermore, Evans reported that a well-differentiated type of liposarcoma showed high frequencies of a local recurrence and metastasis. Therefore, they proposed this tumor as a novel entity of adipocyte-derived tumors, namely, atypical lipomatous tumor (ALT) (2). Although there is no clear definition of clinical subtypes, ALT is classified into two subtypes—superficial type, such as a fat-layer origin, and deep type, such as a muscle-layer or a retroperitoneum origin—in accordance with the occurrence sites, as previously described (3). Superficial type ALT is a rare subtype, and a limited number of cases with superficial type ALT have been reported (4–18). However, the detailed clinical characteristics of superficial type ALT remain unclear.

Besides epidermal and dermal tumors, a subcutaneous tumor is not easy to distinguish the superficial appearance because of the covering by dermis and epidermis. Because of the limited number of evidence, it is difficult to distinguish superficial type ALT from benign lipoma by clinical manifestations. Therefore, the detailed clinical characteristics of superficial type ALT have been desired for a long time. To identify the characteristics of subcutaneous tumors by physical examination, atypical lipomatous tumor is one of the representative subcutaneous malignant tumors with the local invaded characteristics, and dermatologists experience the importance of physical examination of this subcutaneous tumor (19). In addition, because of the unfavorable clinical behavior of superficial type ALT, it is assumed that the tumor size tends to be bigger compared with benign lipoma. However, the actual evidence of these characteristics' importance in superficial type ALT are largely unclear.

Because of the characteristic of subcutaneous tumors, an MRI examination is helpful for the diagnosis. In fact, deep type ALT has a higher positivity of the septal structures in the tumor, which is one of the distinguishing findings from benign lipoma. However, these findings of superficial type ALT have not been reported yet.

One of the most important issues is that little is known about superficial type ALT and it is not easy to predict the presence of superficial type ALT before surgical resection. Deep type ALT shows unfavorable clinical behavior because of high frequencies of local recurrence and high mortality rate. Therefore, initial treatment, especially surgical resection, is a prognostic determinant for ALT patients, and how to predict the presence of ALT as a differential diagnosis is required for clinicians. However, the knowledge of clinical characteristics of ALT is largely unknown.

In this study, we aimed to predict a possible diagnosis of superficial type ALT by physical and MRI examination before surgical resection. We examined 15 cases with superficial type ALT to clarify the detailed clinical characteristics. Our study suggests that the combinations of the clinical characteristic information and MRI are helpful for the diagnosis of superficial type ALT in pre-operation.

## Materials and Methods

### Patients

All 15 superficial type ALT patients, who had already been made the diagnosis at the University of Occupational and Environmental Health, were enrolled in this study from April 1995 to March 2017. The diagnosis of ALT was based on histological assessment according to the WHO criteria (20–22), and all surgical specimens were reviewed and diagnosed by two pathologists who were experienced in the diagnosis of soft-tissue tumors, as previously described (23). In brief, the subjects of the study were histologically characterized by the presence of atypical spindle or pleomorphic stromal cells within often thickened fibrous septa, associated with or without mono- or multivacuolated lipoblasts (23, 24). ALT is classified into two subtypes—superficial type, such as a fat-layer origin, and deep type, such as a muscle-layer or a retroperitoneum origin—in accordance with the occurrence sites, as previously described (3). Clinical characteristics and laboratory examinations, including age, sex, histories, tumor location, tumor diameter, and MRI findings, were investigated in this study. The patients were categorized into two age groups: younger than 60 years and 60 years or older; the presence of symptom: negative group and tenderness or palsy group; the presence of texture: elastic soft and soft; the mobility condition of tumor: good and poor; and tumor size: <4 and 4 cm or longer. We also categorized the location of the tumor into 11 different sites: scalp, face, neck (back neck), shoulder, buttocks, back, chest, abdomen, thigh, and arm. We examined the presence of histories, such as hypertension (HT), diabetes mellitus (DM), hyperlipidemia (HL), and malignancy, to analyze a possible contribution of lifestyle-related histories and the relationship with other malignancies. In the physical examination, the presence of symptoms, tenderness or palsy, texture, and tumor mobility was evaluated by the palpitation in each doctor and was evaluated based on the medical record descriptions. The tumor size was evaluated by the maximum diameter of the tumor.

An MRI examination was performed depending on each doctor's decision whether it is necessary to check the detailed anatomical location before a surgical resection based on their physical examinations. The septal structures of roentgenograph findings in an MRI were made by radiologists in a T1-weighted MRI, as previously described (23).

To clarify the clinical differences between superficial type ALT and benign lipoma, 118 cases with benign lipoma were also enrolled in this study. While superficial type ALT was selected in all cases from 1995 to 2017 periods, we limited the selection of all lipoma cases, who received surgical resection in our department from 2005 to 2017 periods.

### Statistical Analysis

All statistical analyses were performed by GraphPad Prism 8.3.0 (GraphPad Software, San Diego, CA), and *P*-value was evaluated using Student's *t*-test, Fisher's exact test, or Pearson's χ^2^ test.

### Study Approval

This study was approved by the ethics committee of the University of Occupational and Environmental Health (approved number: H29-067) according to the Declaration of Helsinki. Because this study was a retrospective cohort study, the opt-out method of obtaining the waiver of informed consent was adopted based on the ethics committee's approval.

## Results

### Clinical Characteristics

**Figure 1:**
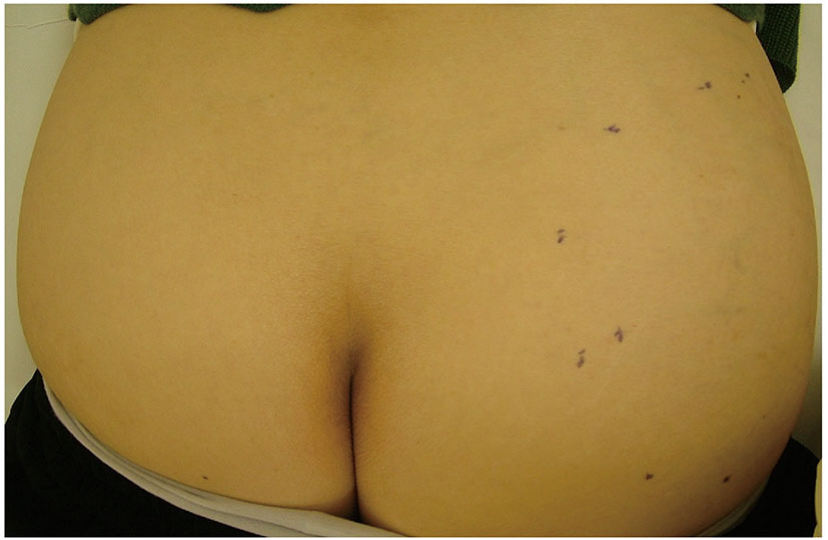
A clinical manifestation of ALT. An elastic soft subcutaneous tumor was located on the right buttocks. Black dotted line indicates the margin of the tumor.

Figure 1 shows a typical clinical manifestation of superficial type ALT in our case. Superficial type is a rare subtype of ALT; however, the clinical characteristics remain unclear. Therefore, we examined the clinical characteristics of ALT, and these characteristics were compared with those of ordinal lipoma because lipoma is one of the most representative differential diagnoses of superficial type ALT. We summarized the clinical characteristics of ALT and lipoma in our study shown in Tables 1, 2. There is a total of 15 cases of ALT and 118 cases of benign lipoma patients in this study. There was no significant difference in age between ALT and lipoma patients. Further, the male:female ratio of superficial type ALT was 2:3, whereas that of lipoma was approximately 1:1. We also analyzed the presence of histories, such as HT, DM, HL, and malignancy. However, there was no significant difference in the frequency of history between ALT and lipoma patients.

**Table 1:** Review of clinical characteristics of superficial type ALT and benign lipoma in our cases.

	**Superficial type ALT**	**Benign lipoma**	***P*-value**
Number cases	15	118	
**Age**			
60 <	8	73	0.580
60>	7	45	
**Sex**			0.785
Male	6	56	
Female	9	62	
**Past history**			
HT	3	24	>0.999
DM	2	8	0.314
HL	0	8	0.596
Malignancy	2	11	0.642
**Symptom**			0.464
Tenderness or palsy	1	20	
Negative	14	98	
**Texture**			0.019
Elastic soft	14	73	
Soft	1	45	
**Mobility**			0.015
Good	11	112	
Poor	4	6	
**Surface color**
Normal	15	118	>0.999
**Tumor size (cm) (mean)**
Range (mean) (cm)	3–20 (9.8)	1–14 (5.0)	<0.001
>4 cm	14	47	<0.001
<4 cm	1	71	
**Septal structure in MRI**			0.0018
Positive	7	9	
Negative	2	35	
Not performed	6	74	

*P-values were evaluated by Fisher exact test or Chi-square test, excluding tumor range, which was evaluated by the student's-t-test*.

**Table 2:** The frequency of detailed location of superficial type ALT and lipoma.

**Location**	**Number (%)**	
	**Superficial type ALT**	**Lipoma**
Scalp	1 (6.6%)	0 (0%)
Face	0 (0%)	10 (8.5%)
Neck (back neck)	2 (13.3%)	22 (18.6%)
Shoulder	2 (13.3%)	8 (6.8%)
Buttocks	2 (13.3%)	5 (4.2%)
Back	2 (13.3%)	38 (32.2%)
Chest	0 (0%)	15 (12.7%)
Abdomen	0 (0%)	5 (4.2%)
Thigh	3 (20%)	4 (3.4%)
Arm	3 (20%)	11 (9.3%)

*P-value was evaluated by Fisher exact test*.

Next, we examined the clinical characteristics based on physical examinations. Clinician experiences to have a difficulty to distinguish the benign tumor and malignancy of subcutaneous tumor. In fact, all these patients with ALT and benign lipoma had no significant difference in the superficial color. In addition, the subcutaneous tumor sometimes causes pain and palsy because the tumor gets pressure surrounding the nerve. However, only 1 ALT patient experienced the tenderness, whereas 20 patients with benign lipoma had palsy or tenderness.

We next focused on tumor growth in ALT. Deep type ALT well-develops in the human body and sometimes presses other tissues, finally leading to other organ dysfunction. In the texture of the tumor, superficial type ALT showed a higher frequency of elastic soft in 93.3% of patients (14 cases), whereas benign lipoma exhibited this finding in 61.9% of patients (73 cases). In addition, because deep type ALT occasionally invades around tissues, it was assumed that this mechanism might also occur in superficial type ALT. As expected, tumor mobility, which is an estimating tool of tumor invasion and adhesion around the tissues, is significantly poorer in superficial type ALT compared with that in benign lipoma. Furthermore, the average tumor diameter in patients with superficial type ALT was 9.8 cm (range of long-axis tumor diameter: 3–20 cm) (*P* < 0.001), whereas benign lipoma was 5.0 cm (range of long-axis tumor diameter: 1–14 cm). Consistently, superficial type ALT showed a significantly higher frequency of the tumor size of more than 4 cm (*P* < 0.001; Table 1). Therefore, the physical examinations for evaluating the tumor size, texture, and tumor mobility are important clinical findings to distinguish superficial type ALT from benign lipoma.

### A Higher Frequency of the Septal Structures in an MRI in Patients With Superficial Type ALT

**Figure 2:**
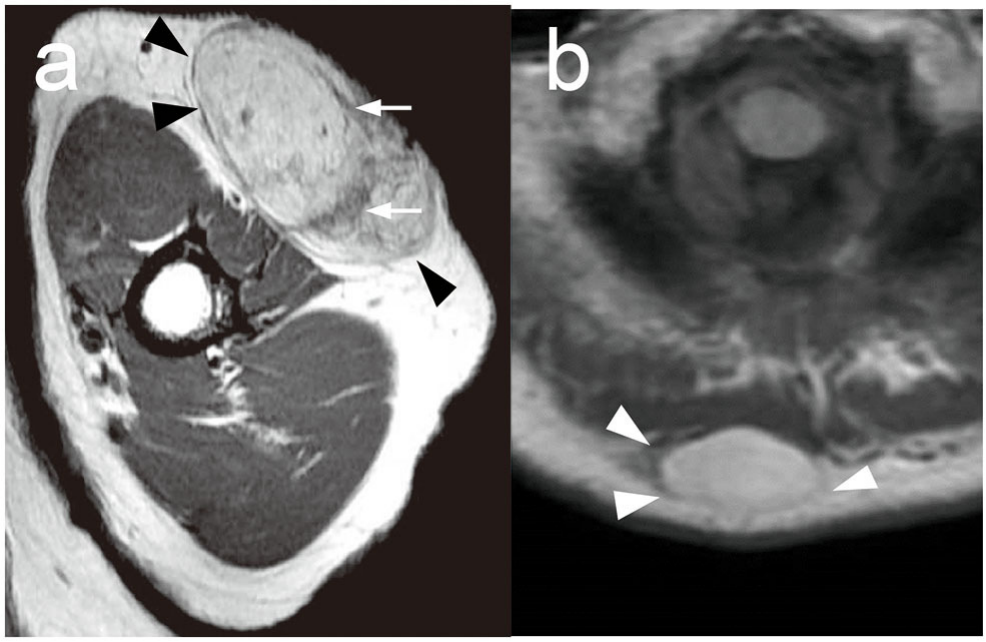
Representative MRI findings in ALT and lipoma. **(a)** A representative MRI of superficial type ALT in the left arm. T1-weighted MRI showed a hyperintense subcutaneous mass with a well-defined smooth margin (arrowheads). Thick hypointense septa (arrows) were recognized. **(b)** A representative MRI of lipoma in the back. T1-weighted MRI showed a hyperintense subcutaneous mass with a well-defined smooth margin (arrowheads) without hypointense septa.

Because subcutaneous tumors could not be distinguished before histological assessment after surgical resection, MRI findings are helpful to determine the therapeutic approaches. A recent study has already identified the usefulness of deep type ALT and other malignant subcutaneous tumors, and MRI findings might also be useful in the diagnosis of superficial type ALT. To clarify this issue, we assessed MRI findings of superficial type ALT and benign lipoma and investigated their characteristics. All cases with superficial type ALT, who received an MRI examination, showed a hyperintense subcutaneous mass with a well-defined smooth margin and thick hypointense septa in a T1-weighted MRI (Figure 2a). Lipoma also showed a hyperintense subcutaneous mass with a well-defined smooth margin (Figure 2b); however, the majority of lipoma patients (79.6%) exhibited no septal structures within the hyperintense subcutaneous mass in T1-weighted MRI. Nine of 15 cases received an MRI examination, and superficial type ALT exhibited 77.8% of cases with the positive septal structures in the tumor and showed a significantly higher frequency of the septal structure findings compared with benign lipoma (*P* = 0.018; Table 1), which was consistent with a previous study (23). Furthermore, one case exhibited a tumor invasive finding around tissue, which is closely related to the physical examination of tumor mobility. From these findings, it seemed that the septal structures in an MRI examination are equally one of the useful findings of superficial type ALT identical to deep type ALT. In addition, it might be rare findings; however, it is useful to estimate the findings of tumor invasion reflecting anatomical interaction of superficial type ALT with surrounding tissues. However, further investigation is necessary to clarify the exact efficacy of these findings because only 9 of 15 patients with superficial type ALT were analyzed in this study.

### The Distribution of Tumor in ALT and Benign Lipoma

To clarify the frequent site of superficial type ALT more precisely, we analyzed the location of these tumors by comparing the differences in the frequency of occurrence sites between ALT and benign lipoma. The tumor locations of superficial type ALT were as follows: trunk, 40% (shoulder, 13.3%; buttocks, 13.3%; back, 13.3%); back neck, 13.3%; extremities, 40.0% (thigh, 20.0%; arm, 20.0%); and scalp, 6.6%. The tumor locations of lipoma were as follows: trunk, 60.2% (shoulder, 6.8%; buttocks, 4.2%; back, 32.2%; chest, 12.7%; abdomen, 4.2%); back neck, 18.6%; extremities, 12.7% (thigh, 3.4%; arm, 9.3%); and face, 8.5%. ALT showed a significantly higher frequency of the presence at extremities (40%) compared with benign lipoma (12.7%). In addition, ALT at the trunk is lower than benign lipoma, especially the back; however, there was a significantly higher frequency of the tumor locations of superficial type ALT in buttocks and shoulder compared with benign lipoma (Table 2). None of the cases with superficial type ALT was seen in the face and front chest and abdomen, whereas lipoma showed frequency with 8.5, 12.7, and 4.2%, respectively (Table 2). Therefore, the high frequent location was different between superficial type ALT and lipoma.

## Discussion

Previous studies have shown that the findings of MRI are useful tools to distinguish deep type ALT from benign lipoma. However, it is valuable for clinicians to have the diagnostic clues of superficial type ALT to distinguish from ordinal lipoma by physical examinations. Our study showed that the evaluation of tumor location and size, texture, and mobility by physical examination is also helpful for a possible diagnosis of superficial type ALT.

Gaskin et al. examined MRI examinations of 126 cases with lipomatous tumors, and ALT exhibited a low intensity in T1-weighted MRI and a high intensity in T2-weighted MRI with the existence of bulkhead structures larger than 2 mm in diameter (25). MRI findings in our case showed the septal structures in the tumor with 77.8% of sensitivity. Therefore, these findings are helpful to make a diagnosis of superficial type ALT in conjunction with the result of the physical examinations.

Several studies have already reported useful information about clinical characteristics in deep type ALT. Deep type ALT showed a significantly higher odds ratio in the tumor size of more than 10 cm (26, 27). On the other hand, our study showed that superficial type ALT had a significantly higher frequency of the tumor size of more than 4 cm. We thought that these findings do not indicate the different tumor characteristics between superficial type ALT and deep type ALT; however, superficial type ALT is easy to be noticed as a subcutaneous tumor in the early time by patients themselves. Because there has been no report focused on the tumor size, these findings are one of the key tools to distinguish superficial type ALT from benign lipoma.

Tumor mobility is also one of the predicting evaluation tools for the subcutaneous tumor to estimate the interaction with surrounding tissue in soft tissue sarcoma (28). Because deep type ALT exhibits a higher frequency of local recurrence rate after surgical resection, it is assumed that superficial type ALT might tend to cause poor tumor mobility by checking physical examination. Our study showed a significantly higher frequency of poor mobility in superficial type ALT, these might reflect the local invasive ability of superficial type ALT. From these findings, clinicians might need to be careful in observing superficial type ALT after surgical resection.

The hardness of the tumor texture contributes to the development of the soft tissue tumor (29). The hardness of the tumor puts pressure on surrounding tissue and easily allows them to expand in the subcutaneous location. Superficial type ALT tends to have elastic hardness compared with benign lipoma, and this might be helpful for superficial type ALT to develop into the subcutaneous location.

Our study also indicates that superficial type ALT showed a higher frequency of tumor site at extremities. In lipoma, the most common locations are the trunk, shoulder, upper arm, thigh, and neck (19, 30). Contrary to these frequent sites of lipoma, superficial type ALT exhibited a significantly higher frequency of extremities. In addition, our study also showed superficial type ALT exhibited a higher frequency of tumor sites at shoulder and buttocks in the trunk. The pathogenesis of ALT remains unclear, and adipose tumor is known to arise from mechanical stress, such as trauma and compression, as the trigger of the tumor (29, 31). One of the most detailed studies on the pathogenesis of post-traumatic lipoma (32) suggested differentiation of mesenchymal precursors to mature adipocytes by trauma. These environmental stimuli might be involved in the pathogenesis of superficial type ALT.

In our study, we could not find significant differences in age, sex, and histories between superficial type ALT and benign lipoma. Several studies indicate that lipoma was observed around 40–50 years old (19, 30). Because of the characteristics of superficial ALT as described previously, it is easy to notice that the tumor might be related with no difference in ages.

We believe that the observation period is a very critical issue to determine the actual recurrence/fatal rate; however, we could not conclude because there was not enough observation period or superficial type ALT patients were transferred to another hospital for the purpose of extended resection. Weiss et al. reported a median time to first recurrence of ALT was 5 to 8 years (retroperitoneal ALT, 8 years; groin ALT, 5 years; extremities ALT, 5 years) (33). To clarify this, it is desirable to investigate their survival and actual local recurrence rate at least over 10 years of observation by conducting a larger-scale study as previously investigated (33).

Taken together, our study showed the importance of physical examination for superficial type ALT and MRI findings. These combined investigations for superficial type ALT are useful for the management of superficial type ALT.

## Data Availability Statement

The raw data supporting the conclusions of this article will be made available by the authors, without undue reservation.

## Ethics Statement

The studies involving human participants were reviewed and approved by the ethics committee of the University of Occupational and Environmental Health. Written informed consent for participation was not required for this study in accordance with the national legislation and the institutional requirements.

## Author Contributions

EM and YS conducted experiment, data analysis, and wrote the main article text. NS-S, KY, SO, DO, HY, MY, and EO performed the treatment and supported data collection. TA prepared MRI figures. MH supported histological analysis. MN wrote the main article, supervised, and organized this study. All authors contributed to the article and approved the submitted version.

## Conflict of Interest

The authors declare that the research was conducted in the absence of any commercial or financial relationships that could be construed as a potential conflict of interest.
